# Abnormalities of AMPK Activation and Glucose Uptake in Cultured Skeletal Muscle Cells from Individuals with Chronic Fatigue Syndrome

**DOI:** 10.1371/journal.pone.0122982

**Published:** 2015-04-02

**Authors:** Audrey E. Brown, David E. Jones, Mark Walker, Julia L. Newton

**Affiliations:** 1 Institute of Cellular Medicine, William Leech Building, Medical School, Newcastle University, Newcastle upon Tyne, United Kingdom; 2 Newcastle Hospitals, NHS Foundation Trust, Newcastle upon Tyne, United Kingdom; 3 Institute for Ageing and Health, Campus for Ageing and Vitality, Newcastle University, Newcastle upon Tyne, United Kingdom; University of Birmingham, UNITED KINGDOM

## Abstract

**Background:**

Post exertional muscle fatigue is a key feature in Chronic Fatigue Syndrome (CFS). Abnormalities of skeletal muscle function have been identified in some but not all patients with CFS. To try to limit potential confounders that might contribute to this clinical heterogeneity, we developed a novel *in vitro* system that allows comparison of AMP kinase (AMPK) activation and metabolic responses to exercise in cultured skeletal muscle cells from CFS patients and control subjects.

**Methods:**

Skeletal muscle cell cultures were established from 10 subjects with CFS and 7 age-matched controls, subjected to electrical pulse stimulation (EPS) for up to 24h and examined for changes associated with exercise.

**Results:**

In the basal state, CFS cultures showed increased myogenin expression but decreased IL6 secretion during differentiation compared with control cultures. Control cultures subjected to 16h EPS showed a significant increase in both AMPK phosphorylation and glucose uptake compared with unstimulated cells. In contrast, CFS cultures showed no increase in AMPK phosphorylation or glucose uptake after 16h EPS. However, glucose uptake remained responsive to insulin in the CFS cells pointing to an exercise-related defect. IL6 secretion in response to EPS was significantly reduced in CFS compared with control cultures at all time points measured.

**Conclusion:**

EPS is an effective model for eliciting muscle contraction and the metabolic changes associated with exercise in cultured skeletal muscle cells. We found four main differences in cultured skeletal muscle cells from subjects with CFS; increased myogenin expression in the basal state, impaired activation of AMPK, impaired stimulation of glucose uptake and diminished release of IL6. The retention of these differences in cultured muscle cells from CFS subjects points to a genetic/epigenetic mechanism, and provides a system to identify novel therapeutic targets.

## Introduction

Chronic Fatigue Syndrome (CFS) is a debilitating condition that affects approximately 600,000 people in the UK [1]. To date, little progress has been made in terms of identifying aetiological processes in CFS. This failure to elucidate key mechanisms has impaired the development of successful diagnostic and therapeutic approaches for the management of CFS.

Skeletal muscle fatigue is a key feature of CFS, and recent studies point to abnormalities of muscle function in those with CFS [2, 3] with similar findings in fatigue associated chronic diseases [4]. Using novel muscle magnetic resonance spectroscopy techniques studies have shown that when CFS patients exercise some generate large amounts of acid within their muscles and have difficulty removing acid when they finish exercising [2, 3]. However, the response to exercise in patients with CFS is heterogeneous with both a variable engagement with exercise and a variable metabolic response [2, 3, 4]. So while there is some evidence of a muscle specific defect, no clear-cut, consistent abnormality has been found. In order to address this, we have devised an exercise system to examine the metabolic response of cultured skeletal muscle cells *in vitro*. In this way we are able to study muscle cell function under standardised conditions that remove the effects of potential confounders encountered *in vivo* that can affect the engagement with and response to exercise.

In recent years, a number of papers have been published describing the development of a method of inducing contraction in skeletal muscle cells using electrical pulse stimulation (EPS). In C2C12 mouse skeletal muscle myotubes, EPS has been shown to accelerate de novo sarcomere assembly via the induction of Ca^2+^ transients [5]. In this model, EPS has also been shown to activate AMP kinase (AMPK), increase glucose transport and enhance the release of chemokines including IL6 [6]. More recently, EPS has been described in human primary skeletal muscle myotubes. Enhanced sarcomere assembly, AMPK activation, increased glycolysis and glucose uptake and increased chemokine expression are key features of this model [7, 8] Taken together, these data indicate that EPS is an appropriate model for examining exercise-related responses in cultured cells.

In the current study, we aimed to use electrical pulse stimulation to examine muscle function using cultured skeletal muscle cells from patients with CFS and healthy controls. The muscle cell cultures are derived from the satellite cells isolated from a needle muscle biopsy sample. The isolated cells first form mononuclear myoblasts and can then be differentiated into multinucleated myotubes that express key characteristics of mature native muscle [9]. An attraction of using the muscle cell cultures is that they are subject to the same standardised conditions, so that any differences that emerge between the CFS and control cultures will reflect changes retained in the cultured cells that are therefore likely to have an epigenetic/genetic basis.

## Research Design and Methods

### Study Subjects

Muscle biopsies were obtained from ten patients diagnosed with chronic fatigue syndrome and 7 healthy control subjects. Groups were matched for age and comprised males and females. All subjects were recruited via the Newcastle NHS CFS Clinical Service at the Newcastle Hospitals NHS Foundation Trust. All subjects fulfilled the Fukuda criteria [10] and provided written informed consent. None had evidence of neurological deficit based on clinical assessment. The study was approved by the Newcastle and North Tyneside Joint Ethics Committee.

### General chemicals and reagents

Cell culture media was obtained from Lonza. FBS and trypsin-EDTA were obtained from Life Technologies (Paisley, UK). Chick embryo extract was purchased from Sera Labs International (Sussex, UK). Phospho-AMPK^Thr172^ (40H9) and total AMPKα (F6) antibodies were obtained from New England Biolabs (Herts, UK). Anti-myosin, skeletal fast (clone MY-32) and β-actin (clone AC-15) antibodies were purchased from Sigma. Monoclonal mouse anti-human desmin (D33) antibody was obtained from DAKO. Vector VIP HRP-substrate kit was obtained from Vector Laboratories. 2-Deoxy-D-[2,6-^3^H]glucose was purchased from Hartmann Analytic (Germany). IL6 ELISA kits were obtained from Qiagen (Sussex, UK).

### Cell culture

Muscle biopsies were obtained from the vastus lateralis and muscle precursor cells isolated according to the method of Blau and Webster [11]. Briefly, needle biopsies were collected in proliferation medium (Ham’s F10 media supplemented with 20% (v/v) FBS, 2% chick embryo extract, 1% penicillin-streptomycin), transferred to a petri dish, washed with PBS and any adipose or connective tissue removed with a scalpel. The tissue was again washed with PBS then cut into small pieces using a scalpel. The tissue was transferred to a universal containing 5ml 0.05% trypsin-EDTA and spin-digested at 37°C. After 15min, the trypsin was removed, 5ml media added and centrifuged at 1700rpm for 5mins. The pellet containing the satellite cells was resuspended in proliferation medium. The spin dissociation protocol was repeated a further 3 times, the pelleted cells were pooled and plated in a T25 flask. Media was changed after 24h to remove unattached cells and cell debris. Cells were expanded in culture and proliferating myoblasts passaged several times before experimentation. Prior to stimulation, cells were seeded at a density of approximately 200,000 per 35mm dish and grown to confluence before inducing differentiation. Differentiation was induced by changing the media to minimal essential media supplemented with 2% (v/v) FBS and 1% penicillin-streptomycin. All experiments were performed on day 7 differentiated myotubes, passage 7.

### Immunohistochemical staining

Muscle cell origin was confirmed immunohistochemically using antibodies to the muscle-specific protein desmin. Cells were fixed and permeabilised by incubation in ethanol at 4°C overnight before washing with phosphate-buffered saline (PBS) and blocking in PBS containing 2% (v/v) FBS. Cells were incubated in anti-desmin antibody at a 1:100 dilution in PBS/FBS 2% for 1hr at room temperature. After washing with PBS and a further incubation in PBS/FBS 2%, rabbit anti-mouse HRP-conjugated secondary antibody was added at a 1:300 dilution for 1hr at room temperature. After washing with PBS, Vector VIP HRP-substrate kit was used to develop the reaction product.

### RNA isolation and cDNA synthesis

Total RNA was extracted from human skeletal muscle cells using the GenElute Mammalian Total RNA Miniprep kit (Sigma) following the manufacturer’s instructions. Briefly, cells were lysed in lysis buffer containing 1% β-mercaptoethanol and applied to a filtration column. An equal volume of 70% ethanol was added to the supernatant and passed through a nucleic-acid binding column. Bound RNA was washed sequentially in Wash buffers 1 and 2. Finally, the column was spin-dried and RNA eluted in a final volume of 50μl. Total RNA was treated with DNase I and 200ng was reverse-transcribed using the High Capacity cDNA reverse transcription kit (Applied Biosystems) in a final volume of 20μl.

### Quantitative real-time PCR

Quantitative real-time PCR was performed on a Lightcycler 480 (Roche) using Taqman primers and probes. MYOG (Hs01072232_m1) was obtained from Applied Biosystems as a predesigned Taqman primer-probe mix and was used at the recommended 1:20 dilution. β2-microglobulin (β2M) was used as a reference gene with sequences: For; GCCTGCCGTGTGAACCAT, Rev; TTACATGTCTCGATCCCACTTACCTATC, Probe; FAM-TGACTTTGTCACAGCCCA-TAMRA. The concentration of both primers was 300nM per reaction and 250nM for the probe. 10μl of Gene expression mastermix (Applied Biosystems) was added to each reaction with 20ng of template. Results were analysed using the standard curve method from a six-point serially diluted standard curve. Reaction efficiencies were 94.3% and 91.5% for MYOG and β2M, with correlation coefficients 0.989 and 0.997 respectively. Relative quantification was performed with data normalised to β2-microglobulin.

### Electrical pulse stimulation

Electrical pulse stimulation (EPS) was performed using a C-Pace EP cell culture pacer (IonOptix, Dublin) using a two-step protocol. Cells were plated in 35mm dishes and when differentiated for 7 days, subjected to EPS at 5volts, 24ms, 2 Hz for 1h followed immediately by 5V, 24ms, 0.2Hz for 1h. This alternation between a period of high frequency and low frequency electrical pulses was continued for the duration of stimulation of 4h, 16h and 24h. Imaging was performed on an Olympus CKX41 microscope and QCapture Pro 6.0 software. Images were taken every 200ms.

### Measurement of lactate dehydrogenase (LDH) release

Lactate dehydrogenase levels were measured in the media from cells subjected to EPS for 4 and 24h using the Lactate Dehydrogenase kit (Sigma). Cells were incubated in fresh media and the media collected at the appropriate time point. LDH release into the media was determined colorimetrically at 490nm according to the manufacturer’s instructions. Briefly, the lactate dehydrogenase assay mixture was prepared by mixing equal volumes of LDH assay substrate, dye and cofactor solutions. Twice the volume of LDH assay solution was added to the media sample and incubated in the dark for 30min at room temperature. The reaction was stopped by the addition of 1N HCl and the colour change read at 490nm. Total intracellular LDH for each cell culture was measured after the addition of 0.9% Triton X-100 and LDH release normalised to total LDH for each cell culture.

### Western blotting

Cells were lysed in extraction buffer (100mM Tris-HCl, pH 7.4, 100mM KCl, 1mM EDTA, 25mM KF, 0.5mM Na_3_VO_4_, 0.1% (v/v) Triton X-100, 1x protease inhibitor cocktail (Pierce)) before sonicating for 10s. Protein concentrations were determined spectrophotometrically at 595nm by a Coomassie binding method (Pierce). 10μg samples were prepared in Laemmli sample buffer (0.125M Tris-HCl, pH 6.8, 4% (w/v) SDS, 20% (v/v) glycerol, 10% (v/v) 2-mercaptoethanol, and 0.004% (w/v) bromophenol blue) and boiled for 5min. After separation on 10% SDS-PAGE gels, proteins were transferred to PVDF membranes using a mini-Hoeffer wet transfer system. After incubation with the appropriate antibodies, detection took place using enhanced chemiluminescence. Phospho-AMPK antibody was used at a 1:1000 dilution while AMPK and myosin were used at a 1:2000 dilution. β-actin was used at 1:10000. Densitometry was performed using a Bio-RAD Molecular Imager GS-800 calibrated densitometer and Quantity One software.

### ELISA

Secretion of IL6 was determined by enzyme-linked immunosorbent assay (ELISA) using the Single-Analyte ELISArray (Qiagen). Skeletal muscle cells were allowed to differentiate for 7 days with media samples being taken at 24h, 72h and 7 days after initiation of differentiation. Cells were subjected to EPS for 4 and 24h 7 days after initiation of differentiation. Cells were incubated in fresh media for the 24h stimulation period. After EPS, media was removed, centrifuged at 1000g for 10min and assayed for secretion of IL6 according to the manufacturer’s protocol. A standard curve was generated by serial dilution of the provided antigen standard and absorbance read at 450nm. Background absorbance was subtracted from the values and the protein concentrations of the samples calculated from the standard curve.

### Glucose uptake

After 16h EPS, cells were incubated in Krebs’ buffer (136mM NaCl, 4.7mM KCl, 1.25mM MgSO_4_, 1.2mM CaCl_2_, 20mM HEPES, pH 7.4) with or without 100nM insulin or cytochalasin B (10μM) for 20min. 0.1mM 2-deoxy-glucose and 0.5μCi (2,6-^3^H) 2-deoxyglucose were added to each well and incubated for a further 10min. The reaction was stopped by washing the plate rapidly in ice cold PBS. Cells were lysed in 0.05% SDS before scintillation counting and protein determination.

### Statistical analysis

All results are expressed as mean±standard error of the mean (SEM) unless where stated. Data were analysed using one-way or two-way ANOVA where appropriate and, where significant, followed up by t-test between groups. Statistical analyses were performed using GraphPad Prism (California) software.

## Results

### Characterisation of the differentiation capacity of skeletal muscle cells

The proportion of desmin-positive cells was calculated after desmin staining by counting the number of desmin-positive cells as a percentage of the total number of cells from 5 fields of view for each cell culture. There were no significant differences in the percentage of desmin-positive cells between the controls and CFS (88.9±7.8% vs 90.7±9.2%, mean±SD, p = 0.5003, respectively).

**Fig 1:**
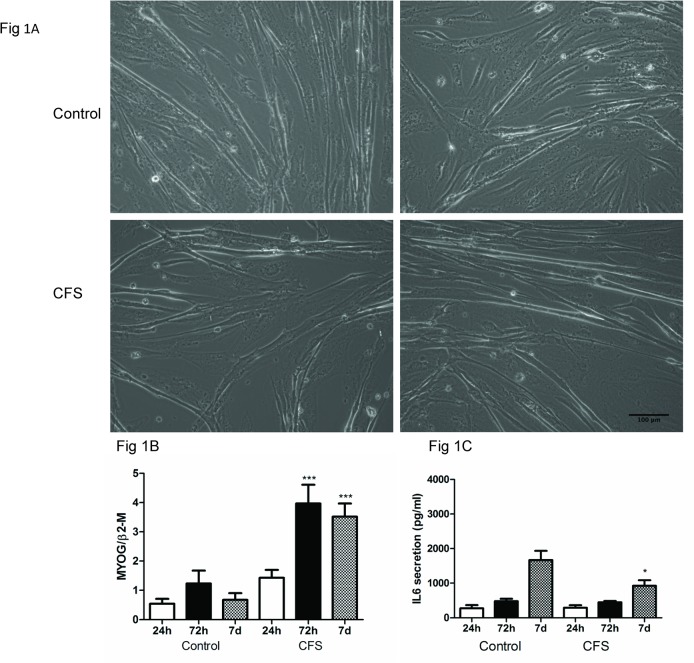
Analysis of the differentiation capacity of control vs CFS cultures. Representative light microscope images of 2 control (top panels) and 2 CFS (bottom panels) cultures taken 7 days after initiation of differentiation. Images were taken at a 10x magnification (A). MYOG expression at 24h, 72h and 7 days after initiation of differentiation from control and CFS cultures was measured by QPCR (B). Data were normalised to the reference gene, β2-microglobulin and are presented as the mean±SEM from 5 controls and 8 CFS subjects analysed in triplicate. ***p<0.0001. IL6 release into the media was measured at 24h, 72h and 7 days after initiation of differentiation from control and CFS cultures by ELISA (C). Data are expressed as the mean±SEM from 5 controls and 8 CFS subjects in duplicate. *p<0.05.

Fig 1A shows representative light microscope images of both control and CFS cultures taken 7 days after initiation of differentiation. Morphological measurements were made from 5 fields of view for each cell culture (Table 1). While there were no statistical differences between control and CFS cultures in terms of length and equidiameter, area was significantly reduced in CFS compared to control (P<0.0001). There was no difference in myotube number between control and CFS cultures at this time point (p = 0.1761). At the 72h time point, while myotube number tended to be higher in the CFS compared with control cultures, this difference was not statistically significant. There were no significant differences in length, area or equidiameter at the 72h time point (S1 Table).

**Table 1:** Morphological measurements were carried out on light microscope images using Nikon NIS-Elements AR Version 4.12 Imaging Software.

Measurement	Control (Mean±SEM)	CFS (Mean±SEM)
Myotube number	24±4.9	23.75±7.6
Length (μm)	392.93±8.13	477.74±8.56
Area (μm^2^)	9792.88±359.77	8006.71±267.94****
Equidiameter (μm)	101.71±1.72	92.86±1.28

Data are from images taken after 7 days of differentiation. Measurements were taken from 5 fields of view for each cell culture, n = 5 cell cultures for control and n = 8 cell cultures for CFS.

****p<0.0001.

A timecourse of differentiation was undertaken to assess the differentiation capacity of cultured skeletal muscle cells derived from patients with chronic fatigue syndrome compared with control cultures. RNA extracts and media samples were taken 24h, 72h and 7 days after initiation of differentiation and analysed for myogenin (MYOG) expression and IL6 release into the media. MYOG is a myogenic regulatory factor required for terminal differentiation of myotubes [12] while IL6 is a cytokine which is produced by skeletal muscle and can play a key role in both muscle cell proliferation and differentiation [13]. MYOG expression was measured by QPCR (Fig 1B) and showed no differences 24h after initiation of differentiation but at both 72h and 7 days, MYOG expression was significantly increased in CFS compared to control (both p<0.0001). Measurement of IL6 release into the media (Fig 1C) indicated that IL6 release was comparable between control and CFS cultures at 24h and 72h after initiation of differentiation but at 7 days, IL6 release was significantly decreased in CFS compared to control (p<0.05).

### Development of an exercise cell culture system

**Fig 2:**
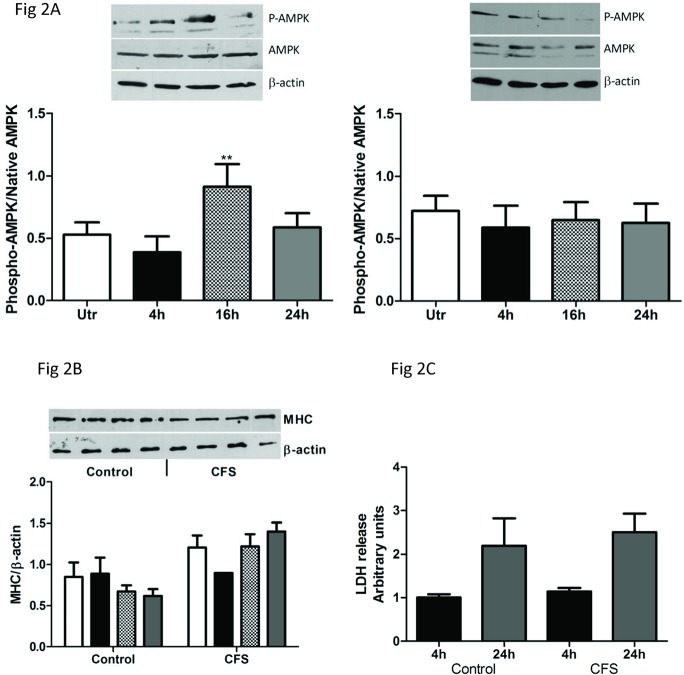
Effect of EPS on AMPK activation, MHC expression and cytotoxicity. AMPK activation was assessed in control (left panel) and CFS (right panel) cell cultures (A). Cells were subjected to EPS and protein extracts made at the appropriate time points. Phosphorylation of AMPK was assessed using phospho-AMPK^Thr172^ antibodies. Densitometry is expressed as a ratio of phosphorylated protein to total AMPK. Open bar: unstimulated, closed bar: 4h stimulation, hatched bar: 16h stimulation, closed grey bar: 24h stimulation. ** p = 0.006 vs unstimulated, Cultures from 7 control subjects and 8 CFS subjects.

Electrical pulse stimulation (EPS) was used to induce contraction in cultured myotubes from healthy controls (S1 Video) and in cultured myotubes from patients with chronic fatigue syndrome (S2 Video). These cultures were subjected to 4, 16 and 24h EPS and examined for metabolic changes associated with exercise *in vivo*. AMP-activated protein kinase (AMPK) is a cellular energy sensor activated by a low energy status such as that seen during exercise. Activation of AMPK was assessed by Western blot using phospho-specific antibodies. In control cultures (Fig 2A, left panel), phosphorylation was shown to increase with EPS and is significantly increased at 16h (p = 0.006). In contrast, chronic fatigue cultures subjected to the same EPS protocol showed no activation of AMPK (Fig 2A, right panel). Expression of the contractile protein myosin heavy chain (MHC) was not altered by EPS (Fig 2B) in either control or CFS cultures with no significant differences in expression between control and CFS at all time points.

Cytotoxic effects of the EPS protocol were assessed by measuring the release of lactate dehydrogenase (LDH) into the media. Fig 2C indicates that LDH release is increased at 24h EPS compared with 4h in both control and CFS cultures. However there are no statistical differences between the two groups at either time point.

MHC protein expression was measured in control and CFS cell cultures (B). Cells were subjected to EPS and protein extracts made at the appropriate time points. Densitometry is expressed as a ratio of MHC to β-actin. Lactate dehydrogenase release (LDH) was measured in control and CFS cell cultures (C). Media from 5 controls and 10 CFS cultures was collected after EPS and assayed for LDH release. Data were normalised to total LDH for each cell culture. Black bar: 4h, grey bar: 24h.

### The effect of exercise and insulin on glucose uptake

**Fig 3:**
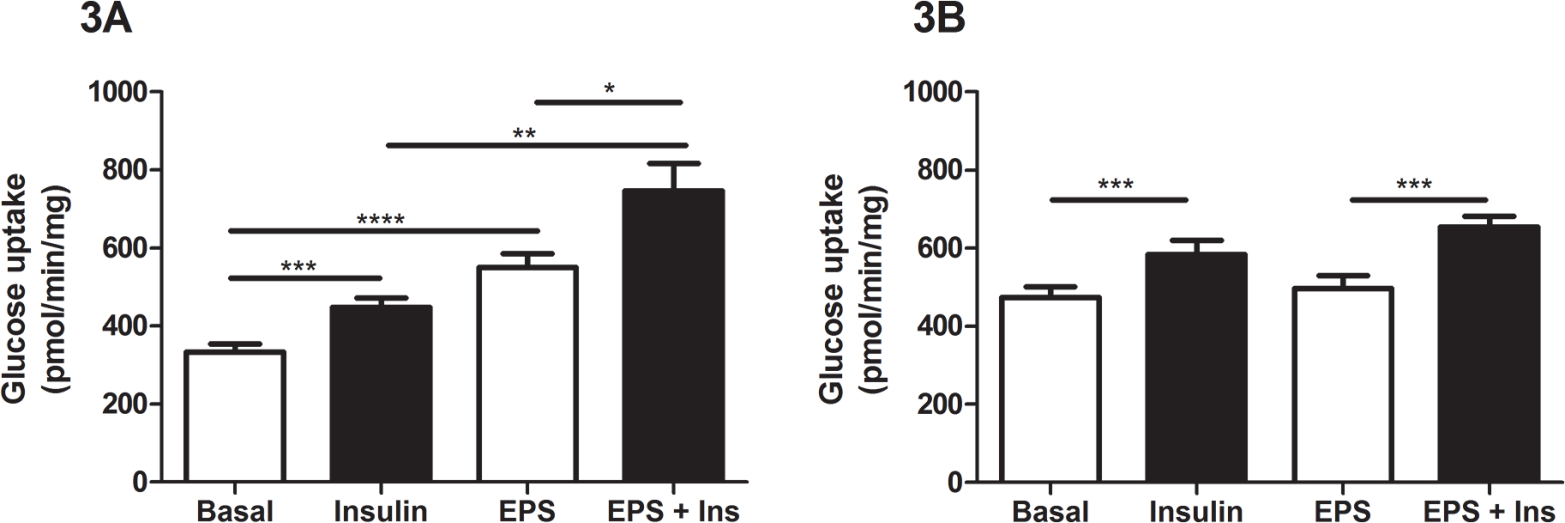
Measurement of glucose uptake in control and CFS cultures. Glucose uptake was measured in control (A) and CFS (B) cell cultures. Cells from 5 control and 9 CFS cell cultures were subjected to EPS for 16h before rate of glucose uptake was measured. Open bar: basal, closed bar: insulin stimulated. Results are expressed as mean±SEM. *p<0.05, **p<0.005, ***p<0.0005, ****p<0.0001.

Contraction increases the uptake of glucose into the muscle cell in an insulin-independent manner therefore, the effect of the EPS protocol on glucose metabolism was examined by measuring uptake of 2-deoxyglucose into the cells in the absence and presence of insulin. In control cells, Fig 3A shows that under non-EPS conditions insulin-stimulated glucose uptake was increased 1.3-fold over basal (447.1±25.3pmol/min/mg vs 333.5±20.2pmol/min/mg, p<0.0005). After 16h EPS, glucose uptake increased to 550.4±35.9pmol/min/mg (p<0.0001 vs basal) while insulin produced an additive effect in combination with EPS, increasing glucose uptake to 746.1±70.9pmol/min/mg (p<0.005 vs insulin alone). Fig 3B shows that under both non-EPS and EPS conditions, the CFS group cultures significantly increased insulin-stimulated glucose uptake (both p<0.0005). However, 16h EPS did not increase glucose uptake in the absence or presence of insulin indicating that the CFS cultures were unable to increase the rate of glucose uptake in response to exercise. This is consistent with the absence of activation of AMPK. Both basal and insulin stimulated glucose uptake were significantly increased in CFS compared to control (p = 0.001 and p<0.05, respectively). However, this does not alter the finding that CFS was unable to increase glucose uptake in response to EPS since an increase in glucose uptake was observed only in the presence of insulin indicating that the cells are not maximally stimulated in the basal state.

### IL6 secretion after muscle contraction

**Fig 4:**
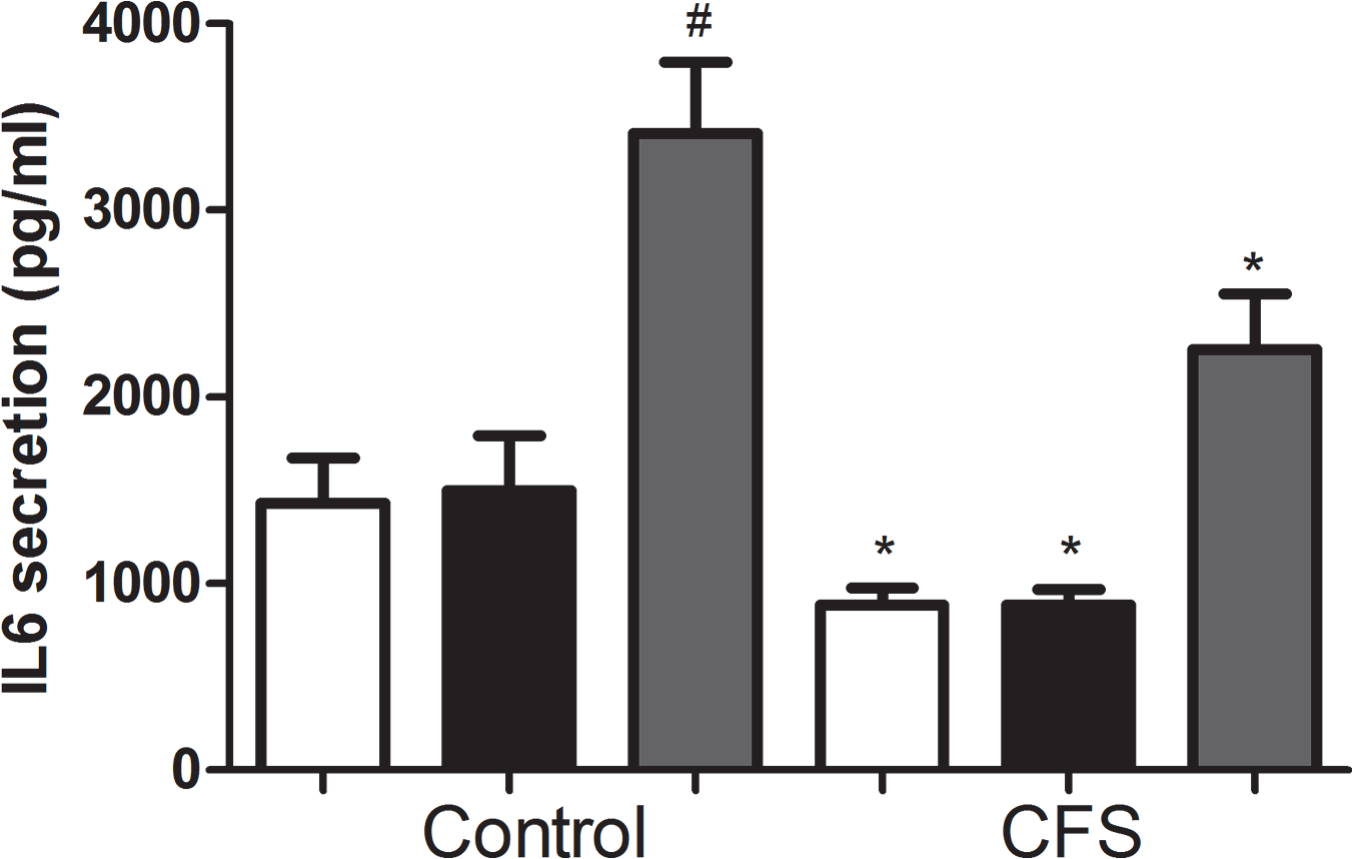
IL6 secretion in control and CFS cell cultures. Fresh media was added to 5 controls and 10 CFS cultures and subjected to EPS for 4 and 24h. After 24h, media was removed and assayed for IL6 secretion by ELISA. Open bar: Unstimulated, closed bar: 4h stimulation, grey bar: 24h stimulation. Results are expressed as mean±SEM. ^#^p<0.001 vs control unstimulated, * p<0.05 vs corresponding control.

Skeletal muscle produces IL6 in response to contraction. The effect of EPS on IL6 secretion is shown in Fig 4. Control cells showed no change in secretion at 4h but was significantly increased at 24h (p<0.001, vs unstimulated). In the CFS group, IL6 secretion showed the same pattern of release but secretion at all time points was significantly decreased compared to control (p<0.05 vs corresponding control), reflecting the reduced basal IL6 release observed previously.

## Discussion

We have used electrical pulse stimulation to generate key aspects of the exercise phenotype in cultured human skeletal muscle cells. Using this method, we showed that electrical pulse stimulation of cultured muscle cells from healthy control subjects resulted in the activation of AMPK and myotube contraction that are characteristic features of exercising muscle. With this exercise model we found two defects in cultured skeletal muscle cells from subjects with chronic fatigue syndrome; impaired activation of AMPK and impaired stimulation of glucose uptake. Interestingly, glucose uptake in response to insulin was normal in the chronic fatigue syndrome cultures, pointing to a specific exercise related defect. The chronic fatigue syndrome and control cultures were studied under identical conditions at passage 7, making it highly probable that the defects in the chronic fatigue cultures are due to retained defects within the cultures of an epigenetic and/or genetic basis.

IL6 release was comparable to controls up to 72h after initiation of differentiation but was significantly decreased in the day 7 chronic fatigue syndrome myotube cultures both before and during contraction. Previous studies have shown no difference in circulating IL6 levels either at rest [14, 15], or after a sub maximal exercise challenge [15] in patients with chronic fatigue syndrome. However, circulating IL6 levels are likely to reflect release from multiple tissue sources. The study we describe here is the first to examine IL6 levels released specifically from skeletal muscle cells from patients with chronic fatigue syndrome. The importance of this decreased IL6 release at the late stage of muscle cell differentiation is not clear. Acute exposure of muscle cells to IL6 and other pro-inflammatory cytokines such as tumour necrosis factor alpha (TNFα) has been previously shown to have a myogenic role in skeletal muscle [13, 16, 17]. Treatment of C2C12 mouse myoblasts for 24h with TNFα in combination with IL6 has been shown to promote myoblast proliferation [17]. IL6 has also been shown to be important in satellite cell proliferation *in vivo* with IL6^-/-^ primary mouse myoblasts showing reduced proliferation in response to muscle overloading [18].

MYOG gene expression was significantly increased at 72h and 7 days after initiation of differentiation in the CFS cultures compared with controls. MYOG is a myogenic regulatory factor which plays a critical role in myoblast differentiation and is a marker of terminal differentiation [19]. MYOG expression tends to increase for the duration of differentiation, peaking at 72–96hr after initiation of differentiation. Increased MYOG expression has been associated with increased IL6 protein release and enhanced myotube formation in cultured C2C12 muscle cells [16, 20]. In the present study, increased MYOG expression was associated with a reduction in IL6 release 7 days after initiation of differentiation in the CFS cultures. Importantly, both control and CFS cultures were differentiated for the same length of time and under standardised conditions before the EPS studies. MYOG expression has been implicated in the exercise capacity of skeletal muscle. An adult mouse model of myogenin deletion showed an enhanced capacity for exercise through the alteration of skeletal muscle metabolism [21]. Compared to their wild-type littermates, MYOG-depleted mice had an increased aerobic capacity through increased oxidative metabolism. It is possible, therefore, that increased myogenin expression may have a negative impact on exercise capacity. Consistent with our findings, an earlier study demonstrated in a mouse model overexpressing myogenin that increased myogenin expression was associated with smaller myofibre area [22].

The lack of activation of AMPK during EPS in muscle cells from chronic fatigue syndrome patients points to a muscle abnormality at the level of, or upstream of, AMPK. AMPK is a heterotrimeric complex composed of a catalytic α subunit and regulatory β and γ subunits. Under normal physiological conditions, AMPK is activated during muscle contraction [23]. Processes, such as muscle contraction, which increase the AMP:ATP ratio promote phosphorylation of AMPK on residue threonine-172 by the binding of AMP (or ADP) to the γ subunit of AMPK. Upstream of AMPK, liver kinase B1 (LKB1) phosphorylates AMPK in most cell types [24]. The Ca^2+/^calmodulin-dependent kinase kinase (CaMKK) can also phosphorylate AMPK in a Ca^2+-^dependent manner in some cells [25]. A more recent study using rat skeletal muscle as a model, suggested that, during moderate muscle contraction, glucose uptake and fatty acid oxidation may be regulated by CaMKK and AMPK via Ca^2+^ signalling [26]. These kinases upstream of AMPK require further investigation in our cohort of CFS subjects and the availability of our *in vitro* muscle system will allow rapid investigation of potential therapies potentially allowing us to fast track optimised treatments to clinical trials.

There is evidence to suggest that IL6 activates AMPK [27]. In skeletal muscle of IL6 knockout mice, after 60min of exercise phosphorylation of AMPK was significantly reduced compared with controls [28]. This study also showed that incubating EDL muscle with IL6 directly increased AMPK phosphorylation. More recently, it has been proposed that IL6 activates AMPK by increasing both cAMP levels and the AMP:ATP ratio [29]. However, the exact mechanism involved is still unknown. In humans, IL6 release from the leg has been shown to correlate significantly with AMPK activity during ergometer cycling [30]. Whether the diminished IL6 release from the CFS muscle cultures contributes to the impaired activation of AMPK warrants further investigation. There is, however, also evidence to suggest that AMPK can regulate IL6 release from skeletal muscle [31].

Electrical pulse stimulation provides an *in vitro* system which allows pre-clinical testing of molecules that could manipulate the biochemical abnormalities seen. Studying those areas of the metabolic pathway that are deficient in patients with CFS and how these might be pharmacologically manipulated would represent an opportunity to ‘fast track’ existing or novel therapies to clinical trials in CFS. In addition to this pre-clinical experimental system allowing us to understand more fully the physiological basis of CFS it could also provide an opportunity to explore fatigue pathogenesis in other fatigue associated chronic diseases. While monolayer cultures provide a good model for studying the response to exercise *in vitro*, a potential limitation of the model is that in 2D cultures the myotubes are not completely aligned. One way to address this is by developing a 3D cell culture system. A number of studies have now been reported where either C2C12 myoblasts [32, 33] or primary rat myoblasts [34–36] have been grown and differentiated in a 3D culture environment which mimics more closely the structural, functional and myogenic properties of native muscle. A recent study has also described the 3D culture properties of primary human skeletal muscle cells [37]. Exercise stimulation in 3D culture is not yet well described but is an important consideration for future experiments.

Recent studies suggest that graded exercise therapy (GET) has benefits for patients with CFS [38] although these benefits have been small. Frequently patients with CFS feel that exercise in fact makes their symptoms worse. Our recent MRS studies suggested that there were at least two muscle phenotypes grouped together through the entirely symptom based diagnostic classification of CFS. This might explain, in part, the limited benefits seen in the PACE trial for GET. We believe that the results of the current study further emphasise the potential for a peripheral component to CFS and the need to fully characterise the muscle phenotypes in CFS before generically prescribing exercise as an effective intervention. Further work is needed to understand the muscle biochemical abnormalities in CFS and the impact that exercise might have upon these.

In conclusion, alternating frequency EPS is an effective model for eliciting muscle contraction and the metabolic changes associated with exercise. The failure of AMPK activation and glucose uptake in response to EPS point to a primary abnormality in the muscle of patients with chronic fatigue syndrome. Mechanisms involved in the activation of AMPK in those with CFS and fatigue associated chronic diseases require further investigation.

## Supporting Information

S1 Table Morphological measurements were carried out on light microscope images using Nikon NIS-Elements AR Version 4.12 Imaging Software. Data are from images taken after 72h of differentiation. Measurements were taken from 3 fields of view for each cell culture, n = 5 cell cultures for control and n = 8 cell cultures for CFS.
(DOCX)

S1 Video EPS-induced contraction of control skeletal muscle cell cultures. Muscle cells from healthy controls were subjected to EPS and imaged using an Olympus CKX41 microscope and QCapture Pro 6.0 software. Images were acquired at x40 magnification.
(ZIP)

S2 Video EPS-induced contraction of CFS skeletal muscle cell cultures. Muscle cells from CFS patients were subjected to EPS and imaged using an Olympus CKX41 microscope and QCapture Pro 6.0 software. Images were acquired at x20 magnification.
(ZIP)
