# Supplementary material for: Abnormalities of AMPK Activation and Glucose Uptake in Cultured Skeletal Muscle Cells from Individuals with Chronic Fatigue Syndrome
Source: PLoS One. 2015 Apr 2;10(4):e0122982. doi: 10.1371/journal.pone.0122982 (PMC4383615; doi:10.1371/journal.pone.0122982)
Supplement: S1 Table — Data are from images taken after 72h of differentiation. Measurements were taken from 3 fields of view for each cell culture, n = 5 cell cultures for control and n = 8 cell cultures for CFS. (DOCX) [file pone.0122982.s001.docx]

|  | Control (Mean±SEM) | CFS (Mean±SEM) |
| --- | --- | --- |
| Myotube number | 14.22±4.17 | 17.91±7.05 |
| Length (µm) | 327.26±12.52 | 398.98±15.06 |
| Area (µm^2^) | 9671.19±956.71 | 8508.59±520.22 |
| Equidiameter (µm) | 102.1±4.66 | 98.81±2.90 |

Supplementary Table 1 Morphological measurements were carried out on light microscope images using Nikon NIS-Elements AR Version 4.12 Imaging Software. Data are from images taken after 72h of differentiation. Measurements were taken from 5 fields of view for each cell culture, n=5 cell cultures for control and n=8 cell cultures for CFS.
