# Supplementary figures and images for: Abnormalities of AMPK Activation and Glucose Uptake in Cultured Skeletal Muscle Cells from Individuals with Chronic Fatigue Syndrome
Source: PLoS One. 2015 Apr 2;10(4):e0122982. doi: 10.1371/journal.pone.0122982 (PMC4383615; doi:10.1371/journal.pone.0122982)

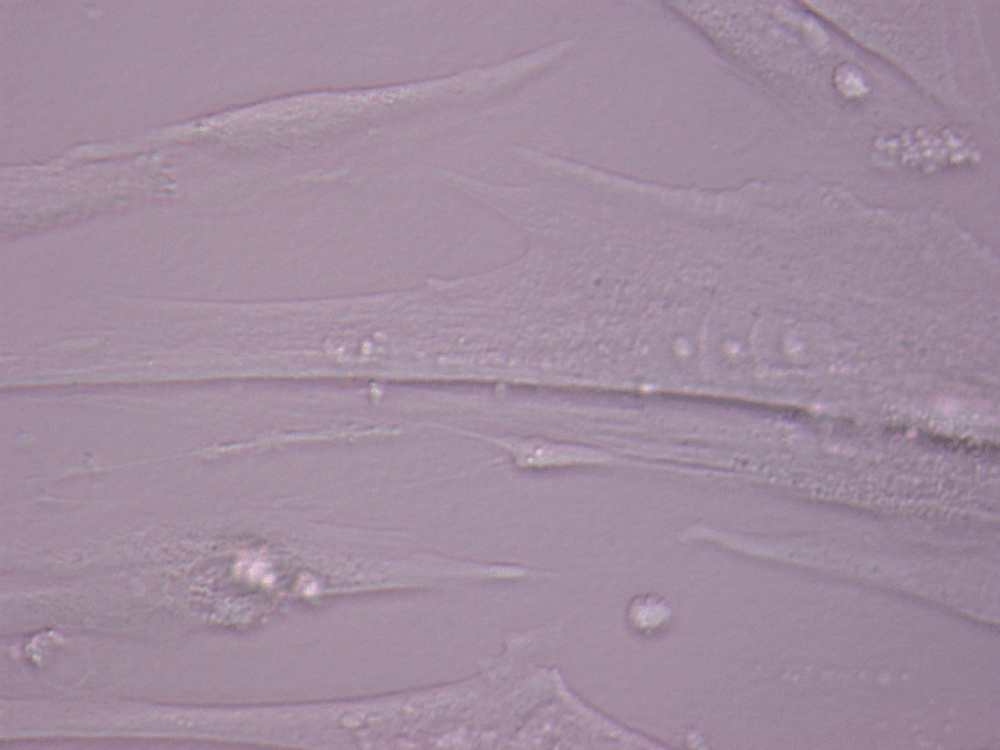

Supplement: S1 Video — Muscle cells from healthy controls were subjected to EPS and imaged using an Olympus CKX41 microscope and QCapture Pro 6.0 software. Images were acquired at x40 magnification. (ZIP) [file pone.0122982.s002.zip › S1 video.gif]

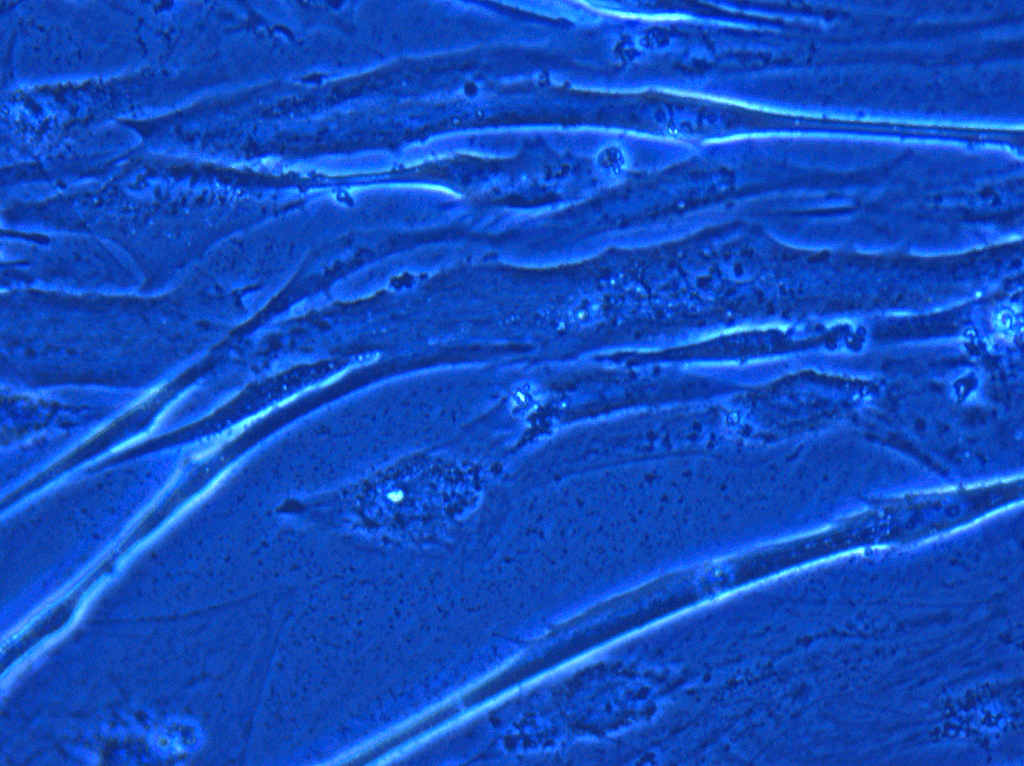

Supplement: S2 Video — Muscle cells from CFS patients were subjected to EPS and imaged using an Olympus CKX41 microscope and QCapture Pro 6.0 software. Images were acquired at x20 magnification. (ZIP) [file pone.0122982.s003.zip › S2 video.gif]
